# Fire Severity and Habitat Type Determine Vegetation Change and Regeneration Time Following Wildfires in Scottish Uplands

**DOI:** 10.1002/ece3.71791

**Published:** 2025-07-15

**Authors:** Noemi A. L. Naszarkowski, Sarah J. Woodin, Louise C. Ross, Alison J. Hester, Robin J. Pakeman

**Affiliations:** ^1^ School of Biological Sciences University of Aberdeen Aberdeen UK; ^2^ The James Hutton Institute Aberdeen UK; ^3^ Rural Land Use Department Scotland's Rural College Aberdeen UK

## Abstract

Wildfire is an increasingly significant disturbance in temperate uplands, yet its long‐term impacts on vegetation remain poorly understood. In Scotland, UK, where blanket bogs and heathlands dominated by *Calluna vulgaris* are widespread, we investigated how wildfire affects vegetation composition and diversity, what the recovery time scales are, and which taxa are most vulnerable. To address these questions, we conducted a space‐for‐time substitution survey across 27 wildfire sites, spanning 2–24 years since fire. Cover of individual plant species and lichens and data on soil carbon, nitrogen, and pH were collected in burnt and adjacent unburnt areas, with fire severity assessed using remote sensing. Regression models were used to examine Shannon diversity, heterogeneity, and compositional dissimilarity between burnt and unburnt areas over time. Effects on community composition were assessed using Canonical Correspondence Analysis (CCA) and Non‐Metric Multidimensional Scaling (NMDS). Our results indicate that wildfire severity and habitat play crucial roles in shaping post‐fire vegetation dynamics. Blanket bog and wet heathland displayed resistance to severe burning, with mild effects on vegetation composition. Dry heathland experienced stronger initial impacts but demonstrated significant recovery over time. Vegetation composition resembled adjacent unburnt areas in approximately 20–25 years following low‐severity fire, and higher‐severity fires prolonged regeneration times. Wildfire reduced Shannon diversity in dry moorland but increased it in wet moorland, with no effect of time since fire. Plot heterogeneity increased with fire severity and also showed no temporal trend. *Sphagnum* abundance showed little relationship with time since fire and may contribute to the resilience of wet moorlands to severe fire. Lichens and pleurocarpous mosses were reduced in cover, whereas graminoids and acrocarpous mosses were abundant in recently burnt areas. The increased risks and consequences of wildfire under climate change may be most severely felt on dry moorland habitats. Although wet moorlands are currently resilient, ongoing management is crucial as future conditions may increase their susceptibility to fire and vegetation change.

## Graphical abstract

This study examines wildfire impacts on vegetation composition and diversity across 27 Scottish blanket bog and heathland sites spanning 2–24 years since fire. Vegetation change and recovery were strongly influenced by habitat type and fire severity, with wet moorlands showing resistance to severe burning and dry heathlands experiencing greater initial impacts but eventual recovery. The findings highlight the vulnerability of dry moorlands under climate change and suggest that, although wet peatlands are currently resilient, ongoing management is crucial as future conditions may increase their susceptibility to severe fire and vegetation change.

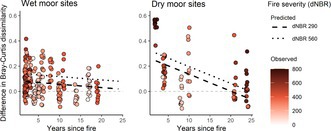

## 1 Introduction

Moorland habitats dominated by *Calluna vulgaris* (hereafter *Calluna*), such as heathlands and blanket bogs, are globally rare but widespread in the UK. These habitats support unique plant and animal species and contain significant carbon stores. Due to warmer and drier conditions driven by climate change, wildfire is an increasing threat to these habitats, with both ignition risk and fire severity rising (Albertson et al. 2010; Lowe et al. 2019). The majority of UK wildfires occur in Scotland, with around 90% of these affecting dry moorlands (dry heath) and wet moorlands (wet heath and blanket bog) (Taylor et al. 2021).

Dry and wet moorlands differ in vegetation composition and fire dynamics. Dry heaths, abundant in pleurocarpous mosses and dwarf shrubs, especially *Calluna*, tend to dry out easily and experience higher fire severity and more pronounced initial impacts on vegetation. In contrast, wet heaths and bogs, characterized by higher graminoid cover and a greater abundance of moisture‐retaining *Sphagnum* mosses (Rodwell 1992), naturally resist burning, reducing severity and vegetation change (Davies et al. 2023; Grau‐Andrés et al. 2018; Naszarkowski et al. 2024; Terrier et al. 2014). However, even wet moorlands can become vulnerable to burning during prolonged dry and warm periods.

Fire severity, often defined as the amount of organic matter consumed above‐ and belowground during a fire (Keeley 2009), influences the magnitude and nature of post‐fire effects on vegetation. Low‐ to moderate‐severity fires can stimulate seed germination and resprouting (Hobbs and Gimingham 1984; Måren and Vandvik 2009). In contrast, high‐severity fires often kill underground stems, deplete seedbanks (Kelly et al. 2016; Mallik and Gimingham 1985; Naszarkowski et al. 2023), and may facilitate colonization by wind‐dispersed species (Schimmel and Granström 1996). Severe fires can also alter soil properties through ash deposition (Pereira et al. 2012), which can inhibit *Calluna* germination (Britton et al. 2003) and facilitate the establishment of species not typical of undisturbed moorland (Pakeman et al. 2012). Although *Sphagnum* mosses are resilient to low‐severity fires (Grau‐Andrés et al. 2017; Hamilton 2000; Whitehead et al. 2021), higher fire temperatures and severity can negatively impact their survival and photosynthetic activity (Noble et al. 2019; Noble, O'Reilly, et al. 2018; Taylor et al. 2017), affecting biodiversity and carbon sequestration. Although vegetation following low‐severity burns usually recovers to its pre‐fire composition and structure within 10–20 years (Harris et al. 2011; Hobbs and Gimingham 1984), very severe fires can create long‐lasting physical barriers, such as hardened peat crusts, that delay recolonization by moorland plants for a longer time period (Clement and Touffet 1990; Maltby et al. 1990; Schimmel and Granström 1996).

Despite the ecological importance and increasing wildfire frequency, long‐term impacts on moorland vegetation and carbon dynamics remain understudied, with most research focusing on immediate post‐fire effects (Davies et al. 2023; Kelly et al. 2016; Shepherd et al. 2021) or extreme fire events (Maltby et al. 1990). Studies on moorland fire dynamics are largely based on prescribed burning, a common management tool in the UK, but these fires are smaller and less severe than wildfires, making direct comparisons difficult.

Beyond climate change and wildfire, Scottish moorland ecosystems face numerous other pressures, including pollution and grazing, which can drive long‐term vegetation shifts (Ross et al. 2012) and potentially interact with wildfire. Grazing by deer and sheep typically reduces dwarf shrubs and mosses while promoting graminoids and may lower wildfire severity by reducing fuel load (Grant and Hunter 1968; Noble, Palmer, et al. 2018; Pakeman and Nolan 2009). Grazing pressure varies across Scotland, making it a crucial factor to include in wildfire studies.

The present study aims to assess moorland resilience to wildfire by asking the following questions:

1. How extensive are the changes in vegetation composition following wildfire, what is the timescale of recovery, and how are these affected by factors such as fire severity, habitat type, topography, and grazing pressure?
2. Do key taxa, particularly *Sphagnum*, show systematic increases or decreases in abundance over time since wildfire?
3. How do alpha diversity (quadrat‐level diversity) and beta diversity (within‐plot heterogeneity) respond to wildfire, and are these responses influenced by time since fire, fire severity, or other environmental variables?

To address these questions, we recorded vegetation at 27 Scottish locations affected by wildfires 2–24 years earlier, comparing burnt areas with adjacent unburnt controls. We hypothesized that initial wildfire impacts would be substantial, with higher severity fires in dry heaths producing greater immediate changes in the vegetation community (Davies et al. 2016, 2023). We expected wildfire, especially at higher severities, to reduce alpha diversity due to species mortality (Davies et al. 2023), while increasing beta diversity by promoting more stochastic recolonization processes and leading to greater heterogeneity in vegetation composition across the landscape (Benscoter and Vitt 2008; Grau‐Andrés et al. 2019; Velle et al. 2014). Species composition and diversity were expected to gradually return to pre‐fire levels over time (Benscoter and Vitt 2008). Given the vulnerability of *Sphagnum* to higher fire severity, we predicted negative impacts of fire on *Sphagnum* abundance followed by slow recovery post‐fire. Although the space‐for‐time approach can be problematic (Damgaard 2019), especially given ongoing large‐scale environmental changes, it remains the only practical method to address these questions.

## 2 Methods

### 2.1 Wildfire Selection

**FIGURE 1:**
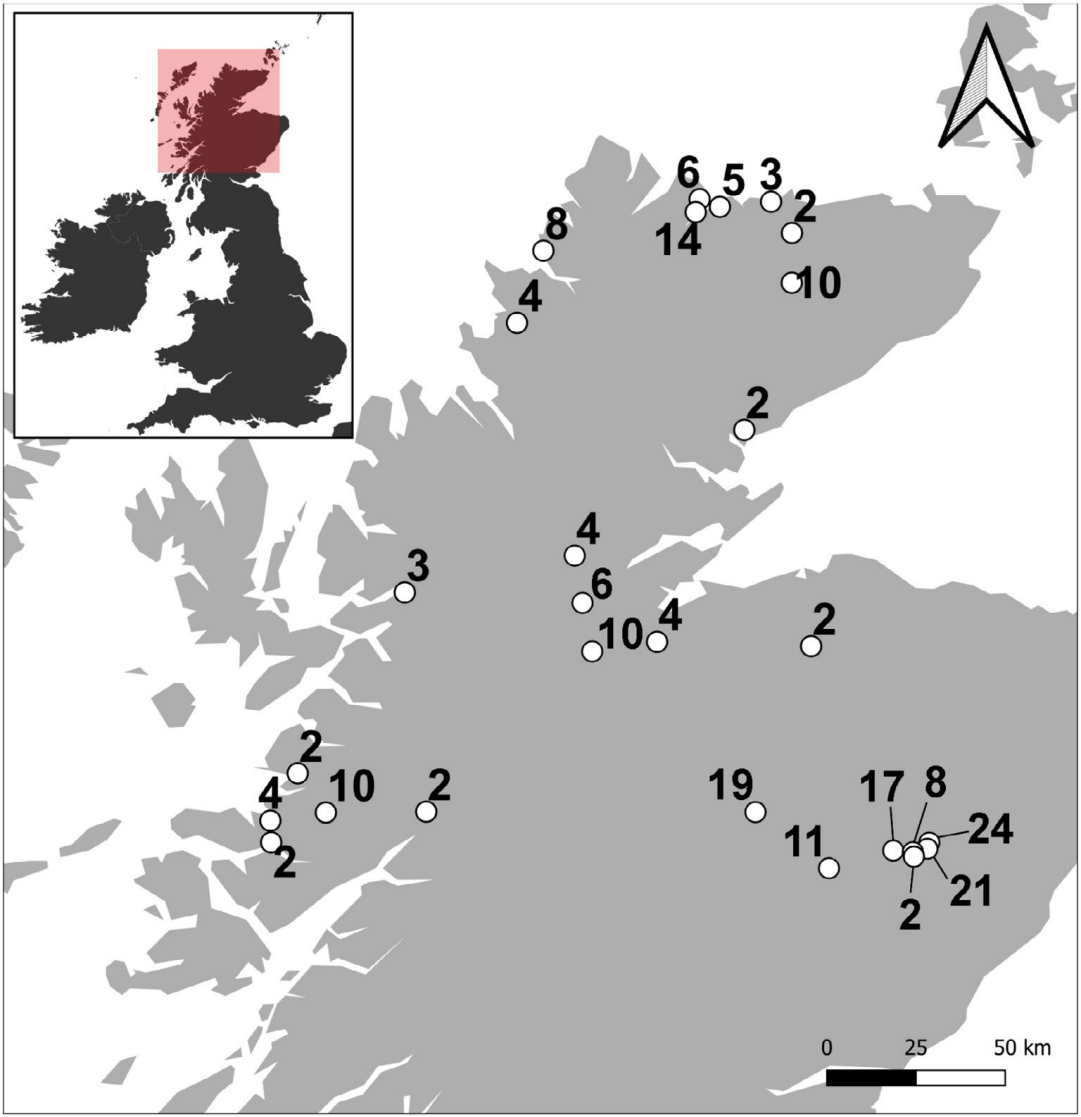
Map showing the surveyed wildfire site locations in Northern Scotland. Labels indicate years since fire at the time of the survey. The inset map provides context, showing the location within Great Britain.

The study was carried out in Scotland, UK, which is characterized by a temperate oceanic climate, with an average total annual precipitation of c. 1600 mm and an annual mean temperature of c. 7.7°C (1990–2020 averages, Met Office 2024). The study exclusively focused on upland wildfires in dry heath, wet heath, or blanket bog, excluding areas dominated by woodland or grassland. Wildfires could include one or a mix of these moorland habitats, but were generally dominated by one. The primary source of wildfire locations was the European Forest Fire Information System (EFFIS), which has mapped European wildfires using remote sensing since 2011. All available wildfire locations in Scotland were downloaded as shapefile polygons from the EFFIS website (EFFIS 2024). To include older wildfires in the survey, additional pre‐2011 wildfires were identified through direct communication with estates and through damage reports from the organizations NatureScot and the Royal Society for the Protection of Birds. The dates and locations of all wildfires were verified using remote sensing. We also reviewed available satellite imagery and fire records for each site to ensure that all selected wildfires represented single burn events, with no evidence of subsequent fires. The objective was to include as many wildfires as possible while capturing a diverse range of fire histories across habitats, all within the constraints of available survey time. A total of 27 wildfires were included in the study (Figure 1).

### 2.2 Sampling Protocol

The survey was conducted in September 2020, June–August 2021, and July 2022. Each wildfire was compared with an adjacent unburnt area. A 1:24,000 scale land cover map (Macaulay Land Use Research Institute 1993), a 1:250,000 scale soil map (Soil Survey of Scotland 1981) and a 5 m resolution digital terrain model (GetMapping 2014) were used to identify comparable burnt and unburnt sampling areas of approximately 10 ha at each site. Comparable areas were defined as moorland of the same type with similar soil type, elevation, slope, and aspect, while prioritizing proximity between burnt and unburnt areas. These areas are hereafter referred to as “plots”, inside which quadrats would be placed, whereas “site” refers to the pair of burnt and unburnt plots linked to each wildfire. We assumed that the unburnt plot at each site was representative of how the burnt plot would appear now, had it not been affected by wildfire. Additionally, we presumed that both burnt and unburnt plots at each site exhibited comparable vegetation composition before the wildfire. We recognize the need for caution in interpreting the results due to inherent limitations of chronosequence methodology, such as site‐specific conditions and disturbance history. Nevertheless, we mitigated these issues by incorporating the site as a random effect in statistical models, as well as by sampling plots within sites in the same visit.

Quadrats were placed in the plots by generating random points using GIS: 10 1 m × 1 m quadrats in the burnt plot at each site and 10 in each unburnt plot. We positioned the plots as closely adjacent as possible, typically 100–300 m apart. However, in instances of limited space, a greater distance between plots was necessary. Moorland habitats are relatively species‐poor and, under similar environmental conditions, tend to support comparable vegetation communities even across substantial distances. The maximum distance between plots at any site was 1200 m, with a mean of 340 m (SD: 353 m) and a median of 200 m (Table 1). Quadrats within a plot were spaced 50–200 m apart and located at least 20 m away from the fire boundary. If a quadrat was located in an area visibly affected by prescribed burning (e.g., a noticeable patch of shorter *Calluna* height), it was excluded, and an alternative random location was chosen. In some instances, limited suitable unburnt plot areas led to a reduction in the number of unburnt quadrats at these sites.

**TABLE 1:** All wildfire sites included in the study, in order of date of fire.

Fire date	Years since fire	Area (ha)	Habitat	Elev.	Lat	Lon	Severity	Plot distance
19/06/1996	24	27	Dry moor	409	57.038	−2.810	602	200
02/04/1999	21	25	Dry moor	442	57.021	−2.814	481	130
12/05/2002	19	70	Wet moor	616	57.036	−3.590	406	100
17/02/2003	17	239	Wet moor	401	56.996	−2.969	106	260
12/04/2007	14	1120	Wet moor	39	58.484	−4.469	179	160
27/04/2011	10	360	Dry moor	249	57.373	−4.509	252	1070
30/04/2011	10	188	Wet moor	150	56.815	−5.553	225	300
18/04/2011	10	315	Wet moor	151	58.365	−3.910	301	280
03/05/2011	11	180	Wet moor	762	56.933	−3.213	401	200
15/09/2012	8	47	Dry moor	402	57.007	−2.879	127	200
22/03/2013	8	108	Wet moor	56	58.314	−5.128	337	160
09/04/2015	6	79	Wet moor	358	57.490	−4.597	244	120
10/04/2015	6	890	Wet moor	58	58.500	−4.463	225	130
10/05/2016	5	442	Wet moor	89	58.513	−4.351	155	550
03/05/2017	4	770	Wet moor	79	56.766	−5.775	92	1100
04/05/2017	4	1550	Wet moor	84	58.138	−5.195	132	1200
15/05/2017	4	47	Dry moor	217	57.413	−4.217	443	530
18/04/2018	3	239	Wet moor	86	58.553	−4.103	397	320
26/04/2018	2	83	Dry moor	552	56.997	−2.865	677	130
28/05/2018	3	497	Wet moor	135	57.413	−5.417	198	380
07/07/2018	4	165	Wet moor	375	57.982	−4.016	489	100
13/04/2019	2	108	Wet moor	213	56.878	−5.098	128	120
19/04/2019	2	124	Wet moor	122	56.901	−5.709	285	150
22/04/2019	2	2718	Wet moor	421	57.452	−3.471	527	130
24/04/2019	2	93	Wet moor	107	56.719	−5.763	354	1140
13/05/2019	2	5430	Wet moor	170	58.427	−4.003	330	120
18/04/2020	2	295	Dry moor	330	57.592	−4.688	401	100

*Note:* Years since fire refers to years since the wildfire event at the time of surveying, which occurred between 2020 and 2022. Area refers to the total burnt area. Habitat type refers to the predominant habitat in burnt and unburnt plots within each site, wet moor indicating blanket bog and wet heath vegetation communities, and dry moor indicating dry heath communities and *Deschampsia* grassland. Elevation refers to mean altitude (m above sea level) of study plots. Latitude and longitude are coordinates of the central point of the burnt plot. Severity is shown as remotely sensed mean difference Normalized Burn Ratio (dNBR) of the burnt plot. Plot distance refers to the distance (m) between the nearest edges of the burnt and unburnt plots.

The 27 sites included in the study comprised a total of 542 surveyed quadrats: 287 burnt and 255 unburnt. A summary of sites is shown in Table 1.

#### 2.2.1 Vegetation Survey and Soil Sampling

In each quadrat, percentage cover of plant species was visually estimated by a single surveyor, recorded to the nearest 5%, with estimates of 1% or 3% used for species with very low cover. Bryophytes and vascular plants were identified to species level. Liverworts often grow hidden in and below the moss layer and were only recorded when they had significant, visible cover, meaning that small and hidden species were omitted. Nomenclature follows Rose (1989) for graminoids, Rose and O'Reilly (2006) for other vascular plants, and Atherton et al. (2010) for bryophytes. Lichens were categorized into only one group, and only those with a foliose (bushy) growth form were recorded, represented mainly by the genus *Cladonia*. One soil sample was collected from below the moss and litter layer of each quadrat using a soil corer of 6 cm diameter and 4 cm depth (113 cm^3^), for analysis of soil bulk density, total carbon, and total nitrogen. At the same depth, a small soil sample was also collected for pH analysis.

### 2.3 Laboratory Analysis of Soil

Soil samples were stored at a maximum of 4°C until they were analyzed. pH analysis was performed within 2 days of collection using a handheld probe. For each sample, a 1:2.5 mixture of soil and deionized water was mixed thoroughly and left for 1 h before measurements were taken.

Soil cores were oven‐dried on aluminium trays for 72 h at 90°C until weight equilibrium was reached and then weighed to estimate soil bulk density. A small subsample of each dried sample was milled to a fine powder, and the total % carbon and nitrogen were analyzed using an NA2500 Elemental Analyser (CE Instruments, Carlo Erba, Milan).

### 2.4 Fire Severity

Fire severity is best measured in the field shortly after the fire event. In this study, sites were visited long after the fire occurred, and therefore, remote sensing using historic imagery was used to estimate fire severity based on the consumption of vegetation. We applied Normalized Burn Ratio (NBR) analysis, an index based on near‐infrared (NIR) and shortwave infrared (SWIR) spectral bands, widely used in forest (e.g., Kurbanov et al. 2022) and non‐forest ecosystems (e.g., Bourgeau‐Chavez et al. 2020). Difference NBR (dNBR), calculated by subtracting post‐fire from pre‐fire values, indicates burn severity, as healthy vegetation has a high NIR and low SWIR response, whereas scorched vegetation shows the opposite (Lutes et al. 2006).

We processed MODIS, Landsat 5, and Sentinel‐2 imagery in Google Earth Engine (Gorelick et al. 2017) using a script from the United Nations Knowledge Portal (United Nations 2017). The spatial resolution of these sensors varies (MODIS: 500 m, Landsat: 30 m, Sentinel‐2: 10 m), affecting severity estimate precision. Sentinel‐2 was used for post‐2015 sites, whereas Landsat was applied to earlier fires. If high cloud cover prevented Landsat use, we relied on MODIS due to its higher temporal resolution. Post‐fire images were taken up to 6 weeks after the fire, and pre‐fire images up to 4 weeks before or from the previous year (±4 weeks). This approach aligns with established remote sensing practices for retrospective fire severity estimation (e.g., Lutes et al. 2006). Fire severity in burnt quadrats, assessed via dNBR, ranged from 18 to 794 (mean = 315, median = 293, 90th percentile = 558).

### 2.5 Grazing

Grazing pressure is challenging to estimate due to limitations in available data. Agricultural census details may lack precision, whereas dung counts during surveys might be too localized and only capture recent visits by grazing animals. Despite observing deer or sheep in most sites during data collection, dung piles were scarce. To address this, we employed agricultural census data for estimating sheep and deer grazing density, offering consistent accuracy across all sites. Sheep grazing was assessed at a 2 km grid resolution (EDINA 2020), and deer at a 1 km grid size (NatureScot 2022). We converted these densities into livestock units using relative size information (Chapman 2017; European Commission 2009), deriving total grazing pressure (livestock units ha^−1^ year^−1^) for subsequent analyses. Livestock units were log‐transformed for statistical analyses.

### 2.6 Vegetation Communities

Using Averis et al. (2004) and TABLEFIT software (Hill 1989), we first classified each site into National Vegetation Communities (NVC; Rodwell 1992). We then simplified this classification into two broad habitat types: wet moor (encompassing blanket bog and wet heath, NVC categories: M2, M6, M15‐M21, and M25) and dry moor (NVC categories for dry heath: H10, H12, H18, H21, and U2).

Initially, we conducted all analyses with three habitat categories: dry heath, wet heath, and blanket bog. However, preliminary multivariate analyses and linear models showed considerable overlap in species composition and post‐fire response between wet heath and blanket bog plots, in contrast to dry heath, which formed more distinct clusters and responses. Based on these results, we combined the wetter habitats into a single group to simplify interpretation and improve statistical power. Despite the mosaic nature of moorland vegetation, broad habitat types were generally consistent within sites. Seven of our sites were classified as dry moor, and 20 sites were classified as wet moor. We acknowledge the potential for vegetation community changes due to fire.

### 2.7 Statistical Analysis

Statistical analyses were performed in R (v. 4.9.2, R Development Core Team, http://cran.at.r‐project.org/) using the package *lme4* (Bates et al. 2015) for regression analyses and *vegan* (Oksanen et al. 2021) for multivariate analyses and calculation of Bray–Curtis dissimilarity and diversity.

#### 2.7.1 Compositional Change and Recovery Time Scales

To assess the impact of fire on vegetation, we calculated the Bray–Curtis dissimilarity between each burnt quadrat and all unburnt quadrats within the same site. The average of these comparisons was used to represent each burnt quadrat, resulting in 287 values included in the analysis. Low dissimilarity values indicate similar composition, whereas high values suggest impact from fire. To establish a baseline dissimilarity expected between two adjacent unburnt areas, we computed the mean dissimilarity between unburnt quadrats within each site. Although this provides an estimate of baseline dissimilarity, the true pre‐fire dissimilarity between plots remains unknown.

We used a linear mixed‐effects model (LMM) to analyze the difference in Bray–Curtis dissimilarity from the mean unburnt baseline. Fixed effects included years since fire, fire severity, and habitat type (wet or dry moor), with site included as a random effect. To examine whether recovery time depended on habitat type, we included an interaction between years since fire and habitat. Because dry moor sites occurred at higher elevations than wet moor sites (mean elevations: 373 m and 290 m, respectively), habitat type was confounded with elevation. To reduce collinearity and improve interpretability, we fitted a second model in which habitat type was replaced with elevation and slope, enabling us to assess whether vegetation recovery was better explained by topographic variables rather than categorical habitat type. We compared models using the small‐sample corrected Akaike Information Criterion (AICc), selecting the one with the lowest value as best supported. We also tested models including a quadratic term for years since fire, but these consistently had substantially higher AICc values and were not selected. Residual distribution was assessed to confirm suitability for a Gaussian model.

To explore quadrat groupings and examine how vegetation change is linked to years since fire and fire severity, we used non‐metric multidimensional scaling (NMDS, function *metaMDS* in *vegan*) based on the Bray–Curtis dissimilarity index. The NMDS was conducted with two and three dimensions and without data transformation. The NMDS with two dimensions had a stress value of 0.22, whereas three dimensions reduced the stress to 0.17. However, we considered the decrease in stress to be insufficient to justify the use of three dimensions, given the challenges of visualizing results beyond two dimensions. Consequently, we utilized NMDS with two dimensions, but due to the stress value exceeding 0.2, caution is advised in interpretation. Results of the three‐dimensional NMDS have been included in Appendices 1 and 2.

#### 2.7.2 Vegetation Composition With Time Since Wildfire

To test the hypothesis that time since wildfire affects the vegetation composition, we used Canonical Correspondence Analysis (CCA), including both burnt and unburnt quadrats. For unburnt quadrats, we assigned a value of 30 years since fire. This was chosen as a conservative estimate based on the typical maximum lifespan of *Calluna* in these habitats, and to avoid unrealistic model behavior due to unknown fire histories or overly long assumed fire‐free intervals. We applied downweighting of species present in < 3% of quadrats to reduce the influence of rare species. Years since fire and environmental predictors (grazing, elevation, slope, aspect, soil bulk density, soil pH, soil N, and soil C:N ratio) were included as predictors, and we used an automatic model selection approach (*ordistep* in *vegan*). We used stepwise, forward selection with 999 permutations to choose the best model. Site and habitat were included as conditional variables, meaning that effects of site and habitat were fitted to the data prior to fitting the predictors, allowing for a more robust test. We used a permutation test (function *anova.cca* in *vegan*) with 999 permutations to test the significance of the predictors.

#### 2.7.3 Wildfire Effects on Diversity

We assessed alpha diversity (species diversity within quadrats) using the Shannon diversity index, which considers the number and relative abundance of species. Beta diversity, indicating compositional variance among quadrats within a plot, was measured using function *betadisper* in *vegan*. For both alpha and beta diversity, we computed a “difference from baseline” variable by subtracting the mean value of unburnt quadrats from the values of burnt quadrats at each site.

Using an LMM, we tested whether plant alpha diversity changed relative to baseline. The site was used as a random effect. Alpha diversity was analyzed at the quadrat level (*N* = 287), and again, we fit one model with habitat and another with topographical variables, selecting the best‐supported model using AICc. Beta diversity, representing heterogeneity among quadrats within a plot, was analyzed as a single average per plot (*N* = 27) using a simple linear model.

## 3 Results

### 3.1 Compositional Change and Recovery Time Scales

Severity had a strong positive effect on dissimilarity between burnt and unburnt quadrats (β ± 95% CI = 0.083 ± 0.043, *p* < 0.001), whereas grazing had no effect (β = −0.001 ± 0.056, *p* = 0.974). There was a non‐significant trend of dissimilarity decreasing with years since fire (β = −0.071 ± 0.071, *p* = 0.065), and a significant effect of habitat, where dissimilarity was higher in dry moor habitat than in wet moor (β = −0.102 ± 0.075, *p* = 0.014). The interaction between habitat and years was marginally significant (β = 0.140 ± 0.129, *p* = 0.051), suggesting that recovery is faster in dry moor habitats. The model explained a marginal amount of variation, with an *R* ^2^ of 0.29 for fixed terms and a conditional *R* ^2^ of 0.55, indicating considerable between‐site variation.

This model, which included habitat rather than topographical variables, had a lower AICc value (−402 vs. −385), indicating it was a more parsimonious model. A model with topographical variables is shown in Appendix 3. In short, elevation had a positive effect on dissimilarity (β = −0.270 ± 0.214, *p* = 0.035), whereas slope had a non‐significant negative effect (β = 0.111 ± 0.111, *p* = 0.089).

**FIGURE 2:**
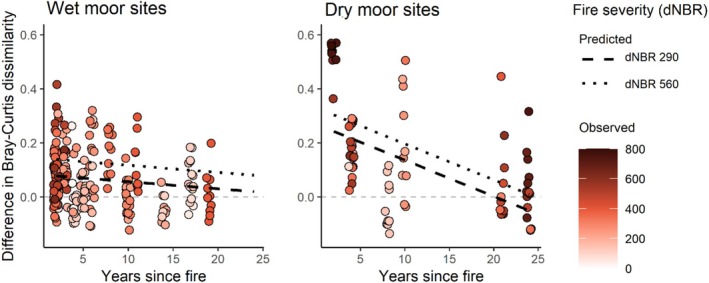
Observed values (points, *N* = 287) and model predicted values (regression lines) of Bray–Curtis dissimilarity between burnt and unburnt quadrats, compared to baseline dissimilarity (dashed grey line), depending on habitat type, years since fire, and fire severity (measured as difference normalized burn ratio, dNBR). Blanket bog and wet heath (left) and dry heath (right) are shown in separate plots for clarity. Black, dashed lines represent dissimilarity at the median severity (dNBR 290), and dotted lines show predictions at high severity (dNBR 560, 90th percentile). Point shading reflects the severity of observed values, with darker shades indicating higher severity. Points are slightly jittered for clarity.

The model predicted that in dry moor habitat, dissimilarity returned to zero after 19 years at median severity and 24 years at high severity (Figure 2). In a wet moor habitat, the dissimilarity increase was smaller than in dry habitat at equivalent severity, but the speed of recovery was slower, and dissimilarity returned to zero after approximately 25 years at median severity and took longer at higher severity. These predictions are limited by the time range of the dataset, and caution is advised due to the marginal significance of the habitat and years interaction (*p* = 0.051). Still, the predictions provide useful insights into recovery dynamics by habitat and fire severity.

**FIGURE 3:**
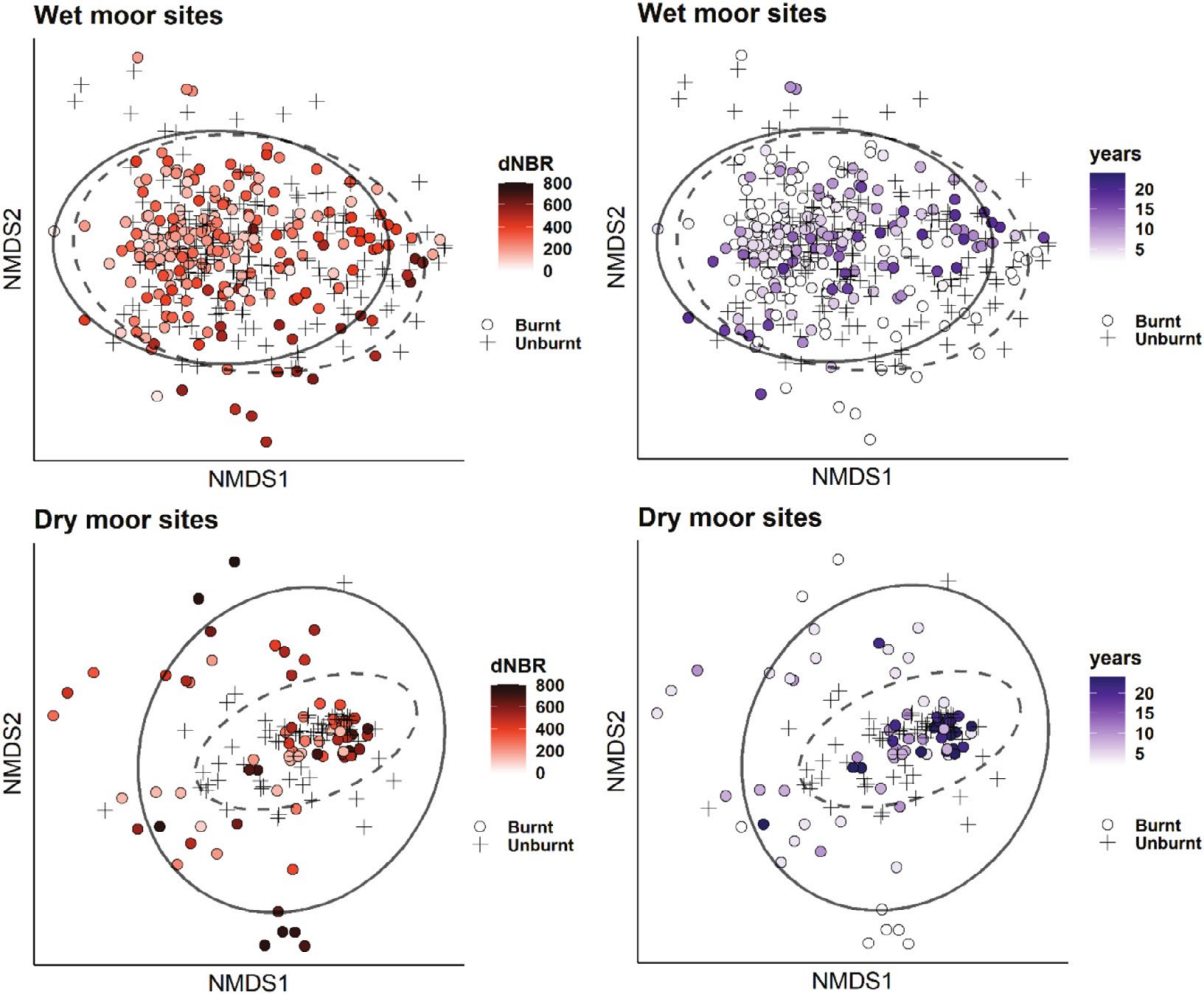
Non‐metric multidimensional scaling (NMDS) showing quadrats affected by wildfire in Scottish uplands. The analysis was performed on all quadrats but is presented separately for wet heath and blanket bog (top, *N* = 397) and dry heath (bottom, *N* = 145), for clarity. Filled circles depict burnt quadrats, with a color gradient depicting years since fire (left), where darker shade of purple equals longer time since fire, and fire severity (right) measured as difference Normalized Burn Ratio (dNBR) where darker shade of red equals higher severity. Crosses represent unburnt control quadrats. Quadrat locations with 95% confidence interval are illustrated with ellipses, where solid line represents burnt quadrats and dashed line symbolizes unburnt quadrats. Stress = 0.22.

Plots for wet and dry sites, including ellipses representing 95% confidence intervals, revealed overlap between burnt and unburnt quadrats (Figure 3). Quadrats with high dissimilarity, situated furthest from the center, were mainly in plots affected by recent wildfire. High fire severity correlated with high dissimilarity, especially in dry moor habitats. High‐severity quadrats near the center tended to be from older fires, suggesting recovery over time, especially in dry moor sites (Figure 3). Lower fire severity was common in wet habitats, contrasting with the relatively high prevalence of high severity in dry moor habitats. NMDS plots with species and with quadrats from wet and dry habitats shown together are found in Appendices 4 and 5.

### 3.2 Vegetation Composition With Time Since Wildfire

**FIGURE 4:**
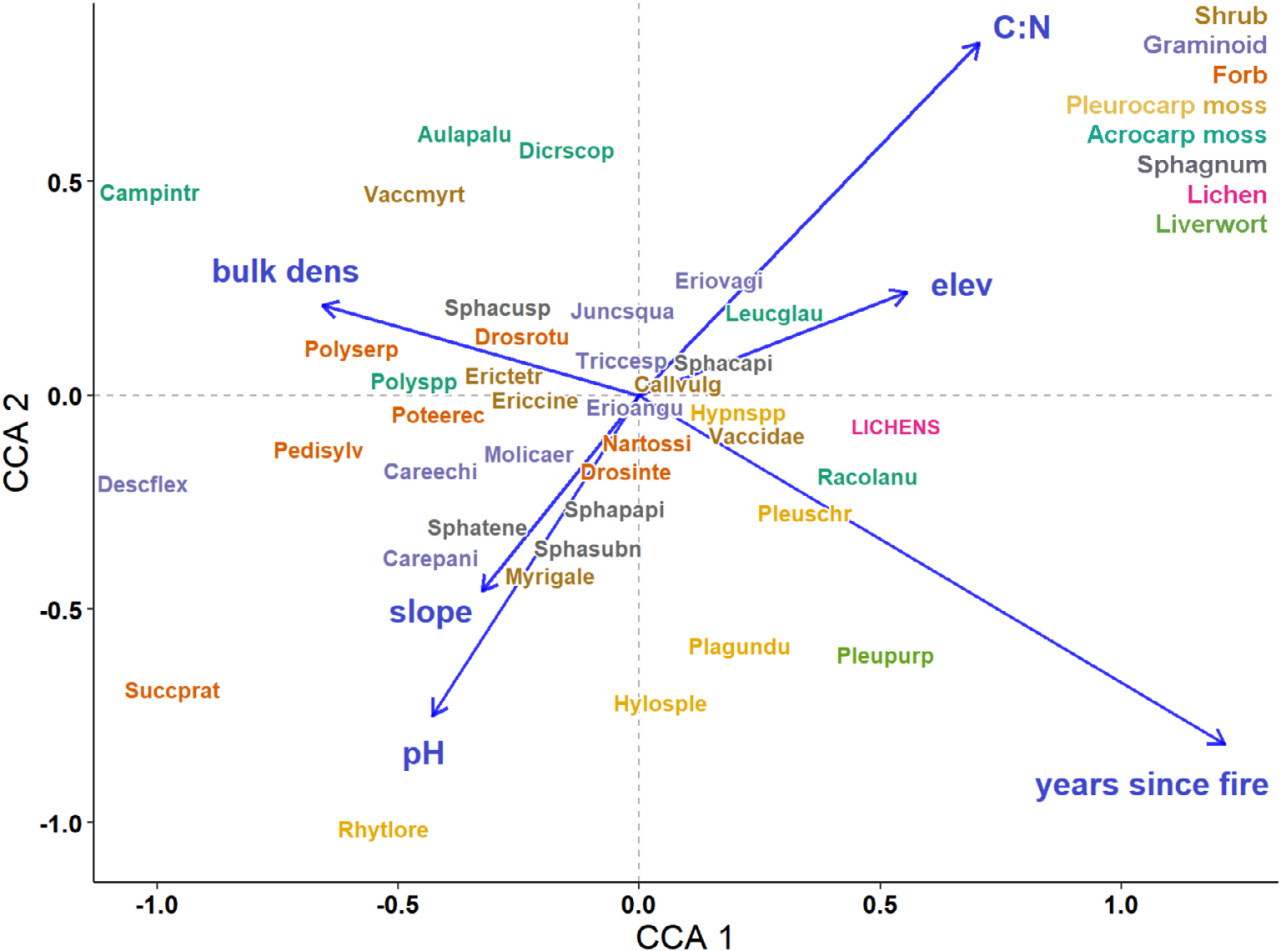
Canonical correspondence analysis (CCA) with years since fire, soil pH, soil bulk density, soil C:N ratio, slope, and elevation as constraints and all species as responses. Site and habitat (wet or dry moor) are conditional effects, meaning they are partialled out before the analysis. Both burnt and unburnt quadrats are included, and unburnt quadrats have been assigned 30 years since fire, whereas time since fire in burnt quadrats is 2–24 years. Functional groups are shown in different text colors. Abbreviations for species names are the four first letters of the genus and species names (full list in Table 2). Only species occurring in > 20 quadrats (out of 542) are included in the plot.

The CCA had 3% constrained variation, 31% conditional variation, and 66% unexplained variation. ANOVA showed that the strongest predictor of species composition was years since fire (*F* = 7.01, *p* = 0.002), and that other important predictors were soil pH (*F* = 3.36, *p* = 0.002), soil bulk density (*F* = 2.56, *p* = 0.004), soil C:N ratio (*F* = 2.28, *p* = 0.006), elevation (*F* = 1.67, *p* = 0.014), and slope (*F* = 1.52, *p* = 0.054). Aspect and grazing were not significant variables (*p* > 0.1) and soil N was excluded from the analysis due to collinearity with C:N, which was the stronger predictor of the two. Bulk density was the only variable that showed a clear correlation with years since fire, and this relationship was inverse (Figure 4).

Many species were situated near the middle of the CCA plot, indicating weak relationships with time since fire, whereas few species stood out as being associated with a long or short time since fire. Most acrocarpous mosses were associated with a short time since fire, most notably *Campylopus introflexus* , but *Racomitrium lanuginosum* was associated with a longer time since fire. Pleurocarpous mosses were linked to a long time since fire, especially *Pleurozium schreberi* , *Hylocomium splendens* , and *Plagiothecium undulatum* , as were lichens and the liverwort *Pleurozia purpurea* . Most *Sphagnum* species showed little dependence on time since fire, though *S. cuspidatum* was associated with a relatively short time since fire. Shrubs generally showed little association, except *Vaccinium myrtillus* , which was linked to very recent fire, and *V. vitis‐idaea* , which was associated with slightly longer time. Forbs and graminoids were either unaffected by or negatively associated with time since fire. The typical heathland pioneer *Deschampsia flexuosa* was the graminoid most strongly linked to recent fire, whereas among forbs, it was *Succisa pratensis* , *Polygala serpyllifolia* , and *Pedicularis sylvatica* . All recorded species and their frequencies by habitat type and fire impact (burnt or unburnt) are shown in Table 2.

**TABLE 2:** Species frequencies in quadrats in dry and wet moorland, in burnt and unburnt plots at wildfire sites.

Abbreviation	Group	Full species name	Dry moorland		Wet moorland	
		Burnt status	Unburnt	Burnt	Unburnt	Burnt
		Total # quadrats	71	74	184	213
Agrocani	Gram	*Agrostis canina*	1 (1)	0	8 (4)	5 (2)
Agrocapi	Gram	*Agrostis capillaris*	0	0	0	1 (< 1)
Agrovine	Gram	*Agrostis vinealis*	1 (1)	2 (3)	0	0
Agrostol	Gram	*Agrostis stolonifera*	1 (1)	0	0	3 (1)
Anthodor	Gram	*Anthoxanthum odoratum*	2 (3)	1 (1)	1 (< 1)	5 (2)
Arctursi	Shrub	*Arctostaphylos uva‐ursi*	0	0	3 (2)	5 (2)
Aulapalu	Acro	*Aulacomnium palustre*	9 (13)	5 (7)	7 (4)	8 (4)
Blecspic	Pteri	*Blechnum spicant*	2 (3)	0	1 (< 1)	1 (< 1)
Breuchry	Acro	*Breutelia chrysocoma*	0	0	5 (3)	4 (2)
Callvulg	Shrub	*Calluna vulgaris*	70 (99)	74 (100)	177 (96)	208 (98)
Campatro	Acro	*Campylopus atrovirens*	0	0	1 (< 1)	3 (1)
Campflex	Acro	*Campylopus flexuosus*	0	0	3 (2)	12 (6)
Campintr	Acro	*Campylopus introflexus*	2 (3)	0	2 (1)	21 (10)
Carebige	Gram	*Carex bigelowii*	0	0	1 (< 1)	2 (1)
Carebine	Gram	*Carex binervis*	2 (3)	4 (5)	1 (< 1)	1 (< 1)
Caredemi	Gram	*Carex demissa*	0	0	1 (< 1)	0
Caredioi	Gram	*Carex dioica*	0	0	1 (< 1)	0
Careechi	Gram	*Carex echinata*	4 (6)	4 (5)	15 (8)	21 (10)
Careflac	Gram	*Carex flacca*	0	0	0	1 (< 1)
Carenigr	Gram	*Carex nigra*	4 (6)	0	3 (2)	6 (3)
Carepani	Gram	*Carex panicea*	2 (3)	3 (4)	13 (7)	19 (9)
Carepauc	Gram	*Carex pauciflora*	1 (1)	1 (1)	2 (1)	1 (< 1)
Carepilu	Gram	*Carex pilulifera*	1 (1)	3 (4)	0	6 (3)
Carepuli	Gram	*Carex pulicaris*	0	0	2 (1)	2 (1)
Carerost	Gram	*Carex rostrata*	0	1 (1)	0	0
Careviri	Gram	*Carex viridula*	0	0	0	1 (< 1)
Cirsarve	Forb	*Cirsium arvense*	0	1 (1)	0	0
Lichens	Lichen	*Cladonia* spp.	37 (52)	28 (38)	84 (46)	54 (25)
Corncana	Forb	*Cornus canadensis*	0	0	1 (1)	0
Dactmacu	Forb	*Dactylorhiza maculata*	0	1 (1)	4 (2)	11 (5)
Dantdecu	Gram	*Danthonia decumbens*	0	0	2 (1)	1 (< 1)
Desccesp	Gram	*Deschampsia cespitosa*	1 (1)	0	0	0
Descflex	Gram	*Deschampsia flexuosa*	8 (11)	17 (23)	5 (3)	6 (3)
Dicrmaju	Acro	*Dicranum majus*	1 (1)	2 (3)	0	0
Dicrscop	Acro	*Dicranum scoparium*	13 (18)	18 (24)	8 (4)	28 (13)
Drosinte	Forb	*Drosera intermedia*	0	0	14 (8)	15 (7)
Drosrotu	Forb	*Drosera rotundifolia*	2 (3)	0	38 (21)	47 (22)
Empenigr	Shrub	*Empetrum nigrum*	2 (3)	0	7 (4)	7 (3)
Equipalu	Pteri	*Equisetum palustre*	1 (1)	2 (3)	0	0
Ericcine	Shrub	*Erica cinerea*	11 (16)	11 (15)	15 (8)	21 (10)
Erictetr	Shrub	*Erica tetralix*	34 (48)	24 (32)	138 (75)	164 (77)
Erioangu	Gram	*Eriophorum angustifolium*	8 (11)	10 (14)	71 (39)	89 (42)
Eriovagi	Gram	*Eriophorum vaginatum*	6 (9)	11 (15)	44 (24)	40 (19)
Euphnemo	Forb	*Euphrasia nemorosa*	0	0	0	1 (< 1)
Festvivi	Gram	*Festuca vivipara*	0	1 (1)	0	2 (1)
Galisaxa	Forb	*Galium saxatile*	3 (4)	1 (1)	0	5 (2)
Geniangl	Shrub	*Genista anglica*	0	0	0	1 (< 1)
Gymncono	Forb	*Gymnadenia conopsea*	1 (1)	0	0	0
Hammpalu	Forb	*Hammarbya paludosa*	0	0	0	1 (< 1)
Holclana	Gram	*Holcus lanatus*	0	1 (1)	0	1 (< 1)
Hupesela	Pteri	*Huperzia selago*	0	1 (1)	2 (1)	1 (< 1)
Hylosple	Pleur	*Hylocomium splendens*	29 (41)	16 (22)	22 (12)	15 (7)
Hypnspp	Pleur	*Hypnum* spp.	49 (69)	32 (43)	73 (40)	92 (43)
Juncbulb	Gram	*Juncus bulbosus*	0	0	3 (2)	0
Junceffu	Gram	*Juncus effusus*	2 (3)	5 (7)	8 (4)	1 (< 1)
Juncsqua	Gram	*Juncus squarrosus*	4 (6)	5 (7)	7 (4)	13 (6)
Lathlini	Forb	*Lathyrus linifolius*	1 (1)	1 (1.)	0	0
Leucglau	Acro	*Leucobryum glaucum*	5 (7)	4 (5)	11 (6)	10 (5)
Listcord	Forb	*Listera cordata*	2 (3)	1 (1)	5 (3)	0
Luzucamp	Gram	*Luzula campestris*	0	1 (1)	0	1 (< 1)
Luzumult	Gram	*Luzula multiflora*	0	2 (3)	0	0
Luzupilo	Gram	*Luzula pilosa*	0	1 (1)	0	0
Lycoclav	Pteri	*Lycopodium clavatum*	1 (1)	0	2 (1)	0
Melaprat	Forb	*Melampyrum pratense*	0	0	0	1 (< 1)
Molicaer	Gram	*Molinia caerulea*	3 (4)	7 (10)	102 (55)	138 (65)
Myrigale	Shrub	*Myrica gale*	0	1 (1)	35 (19)	34 (16)
Nardstri	Gram	*Nardus stricta*	0	0	1 (< 1)	1 (< 1)
Nartossi	Forb	*Narthecium ossifragum*	6 (9)	3 (4)	100 (54)	120 (56)
Pedisylv	Forb	*Pedicularis sylvatica*	0	0	8 (4)	15 (7)
Pingvulg	Forb	*Pinguicula vulgaris*	0	0	4 (2)	3 (1)
Plagundu	Pleuro	*Plagiothecium undulatum*	12 (17)	10 (14)	6 (3)	2 (1)
Pleupurp	Liver	*Pleurozia purpurea*	0	1 (1)	26 (14)	11 (5)
Pleuschr	Pleuro	*Pleurozium schreberi*	18 (25)	8 (11)	33 (18)	11 (5)
Polyserp	Forb	*Polygala serpyllifolia*	0	2 (3)	15 (8.2)	45 (21)
Polyvulg	Pteri	*Polypodium vulgare*	0	0	1 (< 1)	1 (< 1)
Polyspp	Acroc	*Polytrichum* spp.	14 (20)	9 (12)	7 (4)	19 (9)
Poteerec	Forb	*Potentilla erecta*	11 (16)	15 (20)	70 (38)	103 (48)
Pseupuru	Pleuro	*Pseudoscleropodium purum*	0	0	7 (4)	5 (2)
Pteraqui	Pteri	*Pteridium aquilinum*	1 (1)	1 (1)	1 (< 1)	6 (3)
Racolanu	Acro	*Racomitrium lanuginosum*	1 (1)	3 (4)	65 (35)	41 (19)
Rhynalba	Gram	*Rhynchospora alba*	0	0	2 (1)	6 (3)
Rhytlore	Pleuro	*Rhytidiadelphus loreus*	7 (10)	3 (4)	5 (3)	9 (4)
Rhytsqua	Pleuro	*Rhytidiadelphus squarrosus*	4 (6)	2 (3)	3 (2)	2 (1)
Rhyttriq	Pleuro	*Rhytidiadelphus triquetrus*	1 (1)	0	0	0
Rubucham	Forb	*Rubus chamaemorus*	1 (1)	0	6 (3)	2 (1)
Scapgrac	Liver	*Scapania gracilis*	0	0	0	7 (3)
Schonigr	Gram	*Schoenus nigricans*	0	0	0	1 (< 1)
Sphaaust	Sphag	*Sphagnum austinii*	0	0	2 (1)	0
Sphacapi	Sphag	*Sphagnum capillifolium*	16 (23)	8 (11)	102 (55)	111 (52)
Sphacomp	Sphag	*Sphagnum compactum*	0	0	9 (5)	7 (3)
Sphacusp	Sphag	*Sphagnum cuspidatum*	0	0	9 (5)	16 (8)
Sphadent	Sphag	*Sphagnum denticulatum*	0	0	6 (3)	6 (3)
Sphafall	Sphag	*Sphagnum fallax*	1 (1)	0	7 (4)	10 (5)
Sphapalu	Sphag	*Sphagnum palustre*	0	0	2 (1)	3 (1)
Sphapapi	Sphag	*Sphagnum papillosum*	4 (6)	0	32 (17)	45 (21)
Sphasqua	Sphag	*Sphagnum squarrosum*	0	0	1 (< 1)	2 (1)
Sphastri	Sphag	*Sphagnum strictum*	0	0	0	1 (< 1)
Sphasubn	Sphag	*Sphagnum subnitens*	0	0	10 (5)	17 (8)
Sphatene	Sphag	*Sphagnum tenellum*	0	0	22 (12)	28 (13)
Succprat	Forb	*Succisa pratensis*	0	1 (1)	5 (3)	15 (7)
Thuitama	Pleuro	*Thuidium tamariscinum*	0	0	4 (2)	3 (1)
Triccesp	Gram	*Trichophorum cespitosum*	33 (47)	24 (32)	116 (63)	155 (73)
Vaccmyrt	Shrub	*Vaccinium myrtillus*	14 (20)	25 (34)	12 (7)	11 (5)
Vaccidae	Shrub	*Vaccinium vitis idaea*	17 (24)	19 (25)	7 (4)	3 (1)
Verooffi	Forb	*Veronica officinalis*	1 (1)	0	0	0
Violpalu	Forb	*Viola palustris*	0	1 (1)	0	0

*Note:* Frequencies are also shown in brackets as percentages of the total number of quadrats in the corresponding category (e.g., unburnt dry moorland). The table also includes species abbreviations used in Figure 4, and the functional group the species belongs to (gram, graminoid; acro, acrocarpous moss; pleuro, pleurocarpous moss; pteri, pteridophyte; liver, livewort; sphag, Sphagnum moss).

### 3.3 Wildfire Effects on Diversity

**FIGURE 5:**
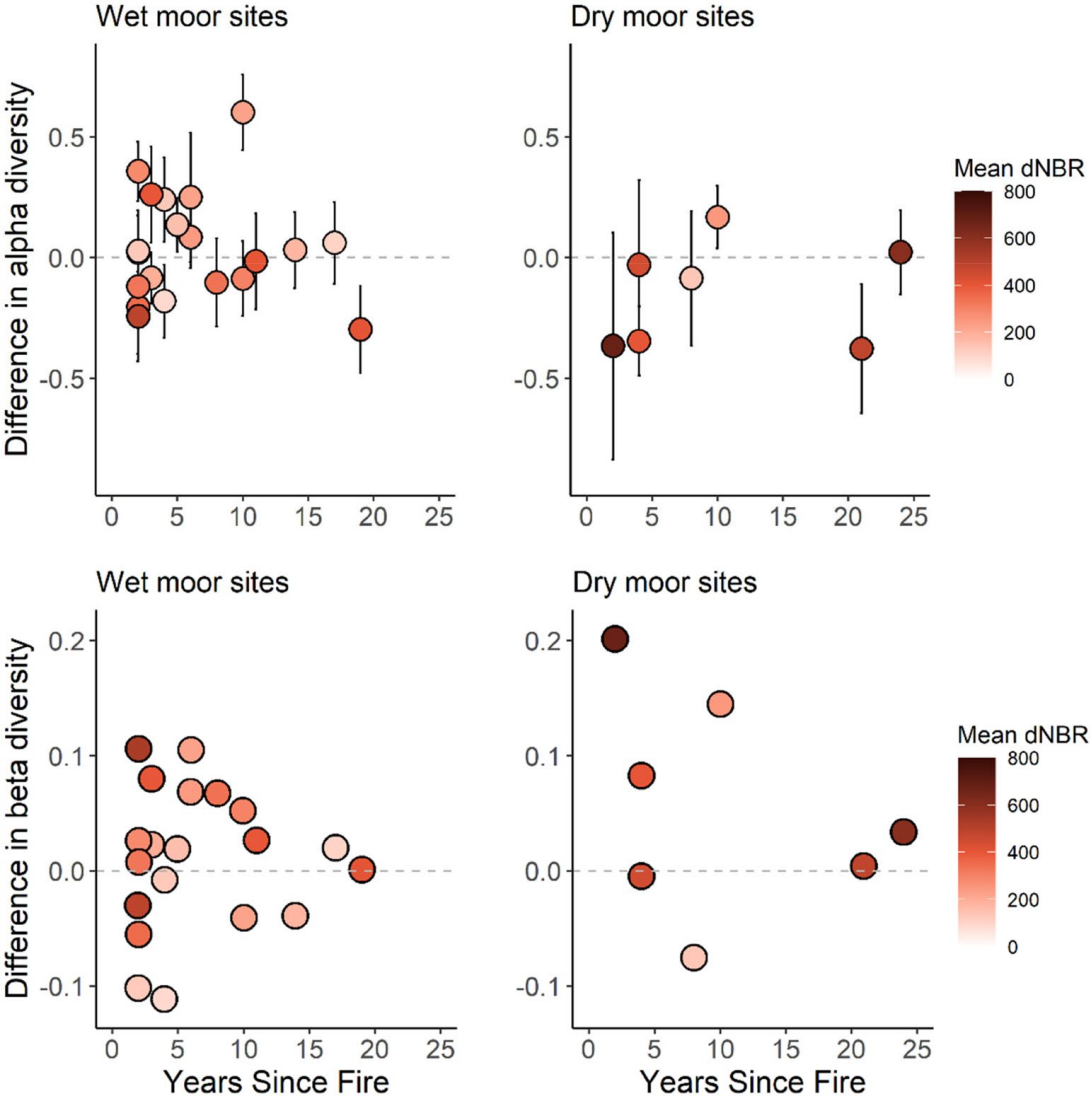
Differences in plant community diversity in wildfire‐affected plots burnt 2–24 years earlier, compared to unburnt control plots (dashed, grey line). The top row shows quadrat‐level alpha diversity, measured as Shannon diversity (mean ± 95% CI, *N* = 287), whereas the bottom row shows mean beta diversity, representing heterogeneity among quadrats within a plot (*N* = 27). Blanket bog and wet heath sites (left) are shown separately from dry heath sites (right) for clarity. Point colors indicate fire severity, measured as the difference Normalized Burn Ratio (dNBR).

For alpha diversity, a model including habitat rather than topographical variables was better (AICc of 243 versus 286). This model had a marginal *R* ^2^ of 0.18 and a conditional *R* ^2^ of 0.40, suggesting low explained variance. Habitat was the only significant predictor in this model (*p* < 0.001, Figure 5, Table 3). The model indicated that alpha diversity was decreased in dry habitat after wildfire compared to unburnt baseline, whereas it was increased in wet habitat, and there was no evidence of this changing with time since fire (*p* = 0.489). Grazing was not significant (*p* = 0.559) and neither was the interaction of years and habitat (*p* = 0.556). A model with topographical variables is shown in Appendix 6. In short, elevation had a significant negative effect on alpha diversity (*p* = 0.018), whereas slope had a significantly positive effect (*p* = 0.050).

**FIGURE 6:**
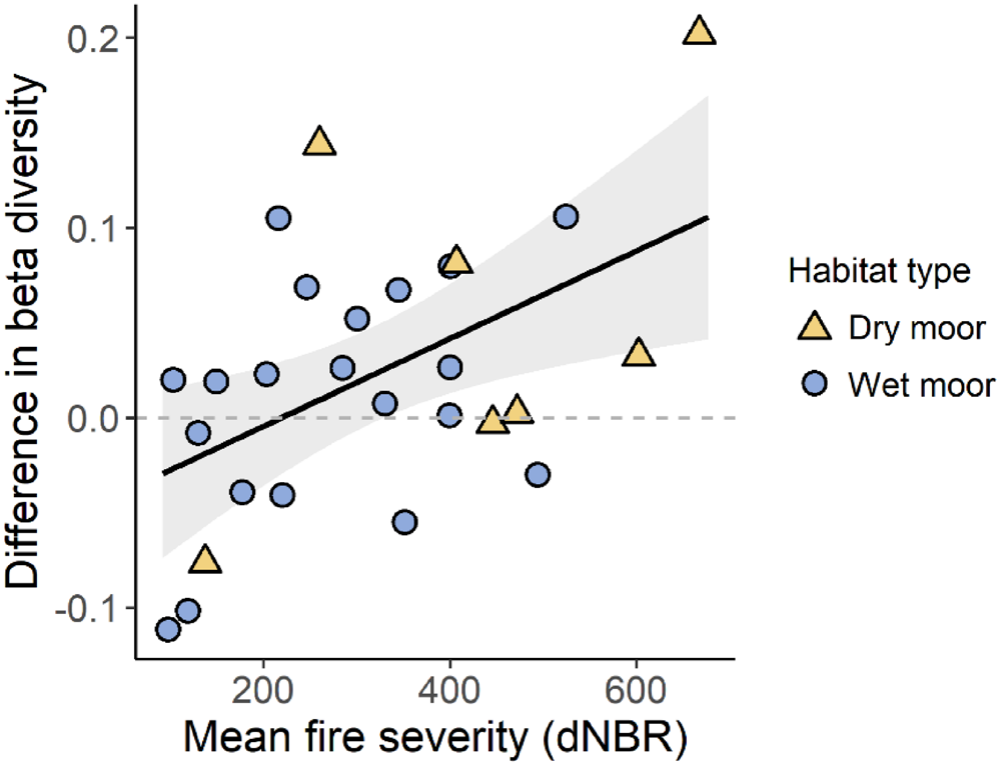
Observed values (points, *N* = 27) and model‐predicted values (regression line) of differences in beta diversity in wildfire‐affected plots compared to the unburnt baseline (dashed grey line), as a function of fire severity (measured as the difference Normalized Burn Ratio, dNBR). Blanket bog and wet heath plots are shown as blue circles, whereas dry heath plots are represented by yellow triangles.

The model predicting beta diversity had an *R* ^2^ of 0.28. Beta diversity was positively correlated to fire severity (*p* = 0.020, Table 3), indicating that high‐severity fires led to greater compositional heterogeneity among quadrats within a plot (Figure 6). However, years since fire had no significant effect (*p* = 0.884). Habitat (*p* = 0.605), grazing (*p* = 0.760), and the interaction between years since fire and habitat (*p* = 0.216) also showed no significant effects. Visually, the data suggest that differences in beta diversity may be more variable, both positively and negatively, in the years immediately following a fire, particularly within the first 10 years (Figure 5). Over time, this variability appears to stabilize closer to the baseline in both wet and dry moorland sites.

**TABLE 3:** Results of linear mixed‐effects model for alpha diversity (Shannon diversity index, *N* = 287) and simple linear model for beta diversity (*N* = 27).

Variable	Estimate ± 95% CI	*t*	*p*
Effects on alpha diversity (Shannon diversity index) (*N* = 287)			
Severity (dNBR)	−0.049 ± 0.127	−0.753	0.453
Years since fire	0.063 ± 0.175	0.702	0.489
Wet habitat	0.368 ± 0.112	−6.435	< 0.001
Grazing	−0.046 ± 0.152	−0.590	0.559
Years since fire × habitat	−0.060 ± 0.200	−0.590	0.556
Effects on beta diversity (*N* = 27)			
Severity (dNBR)	0.077 ± 0.060	2.520	0.020
Years since fire	−0.004 ± 0.057	−0.148	0.884
Wet habitat	−0.018 ± 0.066	−0.525	0.605
Grazing	−0.008 ± 0.054	−0.310	0.760
Years since fire × habitat	0.069 ± 0.106	1.277	0.216

*Note:* Coefficient estimates are standardized. Severity is measured as difference Normalized Burn Ratio (dNBR). Habitat estimates are for wet moor habitat, with dry moor habitat as the reference level.

## 4 Discussion

### 4.1 Compositional Change and Recovery Time Scales

Consistent with our hypothesis, the dissimilarity between vegetation in burned and adjacent unburned areas initially increased after fire, with a significant positive correlation to fire severity. Additionally, as anticipated, our results suggest that regeneration responses may vary among moorland habitats. Dry moors experienced stronger initial impacts, returning to pre‐fire composition after a minimum of approximately 20 years, depending on severity. In contrast, wet moors showed weaker effects, with less change over time, potentially due to the typically lower fire severity associated with wet habitats (Davies et al. 2016; Grau‐Andrés et al. 2018, 2019; Naszarkowski et al. 2024). Comparable recovery times of 20–30 years have been observed in Canadian bogs (Benscoter and Vitt 2008).

Our results align with other studies looking at long time scales, which confirm that severity is a key determinant of speed and direction of successional trajectories after wildfire in bog (Clarke et al. 2015), and that vegetation composition in *Sphagnum*‐dominated habitats is resilient to wildfire (Kuhry 1994). The NMDS diagram illustrated that the most dissimilar quadrats corresponded to recently burned areas (2–4 years) and/or were affected by high fire severity, emphasizing the fundamental role of severity. Notably, quadrats in dry heath impacted by severe fires long ago (21 or 24 years) exhibited low dissimilarity, suggesting long‐term resilience to fire, potentially attributed to rotational burning practices typical in dry moorlands. Anthropogenic fires may have resulted in an increased abundance of fire‐adapted species in dry moorlands, for example, through the evolution of traits such as fire‐induced germination (Bargmann et al. 2014; Vandvik et al. 2014). Our findings underscore the resistance of wet heath and blanket bog to high fire severity, although extreme events during drought may have a more substantial impact on vegetation, leading to slower regeneration. While predictions for wet moorland sites with a long time since severe fire remain uncertain, they align with reports of slow regeneration after extremely severe moorland wildfires (Legg et al. 1992; Maltby et al. 1990).

### 4.2 Vegetation Composition With Time Since Wildfire

As expected, fire was a strong predictor of vegetation composition, and the CCA revealed that years since fire impacted vegetation composition more than environmental variables related to soil or topography. Most quadrats had experienced low to moderate fire severity, while high severity burns were rare, likely explaining the limited signs of severely inhibited regeneration—unlike the more substantial vegetation impacts reported in studies where fire severity was higher (Clement and Touffet 1990; Maltby et al. 1990).

Consistent with trends observed in previous studies, graminoids, forbs, and acrocarpous mosses were associated with recently burned areas, whereas pleurocarpous mosses and lichens exhibited negative impacts, requiring an extended recovery period (Clement and Touffet 1990; Davies et al. 2023; Grau‐Andrés et al. 2019; Hobbs and Gimingham 1984; Schimmel and Granström 1996). Shrub responses varied, with *Calluna* showing weak dependence on time since fire, whereas *Vaccinium myrtillus* exhibited a strong association with short time since fire, as can be expected for this typical pioneer with rhizomatous spread and high growth rates (Davies et al. 2023). Ericaceous shrubs have previously been found to generally increase after recent fire but also to show high variability depending on site, species, and fire severity (Davies et al. 2023; Schimmel and Granström 1996).

One of the aims of this study was to assess the effects of wildfire on *Sphagnum*, as active peat formation in wet moorlands is important for resilience to climate change. Contrary to our expectation that wildfire would be detrimental to *Sphagnum*, the results show little indication that *Sphagnum* mosses as a group are negatively impacted by wildfire. This may be attributed to their high moisture content and resistance to burning (Terrier et al. 2014), their resilience and fast regeneration after low to moderate severity fire (Taylor et al. 2017), and/or the possibility that *Sphagnum* may benefit from reduced competition for space after fire (Lee et al. 2013; Noble, O'Reilly, et al. 2018). Although individual *Sphagnum* species may exhibit varied responses, analyzing this was challenging due to the scarcity of many species. *S. cuspidatum* was the *Sphagnum* species most related to a shorter time since fire, which can be explained by its aquatic habit and presence in bog pools, which may be resistant to drying out and burning. This contradicts earlier research from Canada, which found that *S. cuspidatum* is more damaged by wildfire than *S. capillifolium* (Blier‐Langdeau et al. 2021). This may be explained by the intense drought before the fire in the studied Canadian bog, leading to a substantial drop in water table, and the susceptibility of *S. cuspidatum* and related species to burning in such conditions. Lawn and hummock‐forming species with denser growth habits, like *S. capillifolium* , may better retain moisture, making them more resilient to drying out (Blier‐Langdeau et al. 2021). Because severe bog fires, most likely caused by extreme drought, were rare in our dataset, we did not observe negative effects of fire on aquatic *Sphagnum* species.

The only acrocarpous moss not associated with a short time since fire was *R. lanuginosum* , which may be explained by this species' slow‐growing habit compared to other acrocarpous mosses. It has been noted as sensitive to fire and can be an indicator of declining *Racomitrium* heath communities of high conservation value (Averis et al. 2004; Pearce and Van Der Wal 2002; Ross et al. 2012; Tallis 1995). In contrast, our analysis evidenced a strong association between recently burnt areas and *Campylopus introflexus* . This invasive acrocarpous moss is an aggressive colonizer of bare ground in burnt or eroded moorland areas and can be a potential threat to reestablishment of keystone taxa like *Sphagnum* or *Eriophorum* (Equihua and Usher 1993; Noble, Palmer, et al. 2018). Increased nutrient availability following fire can benefit *C. introflexus* , while simultaneously inhibiting *Sphagnum* growth (Gunnarsson and Rydin 2000; Southon et al. 2012). Predicted higher wildfire severity in moorlands may therefore threaten the establishment of keystone species, due to increased areas of bare peat and abundant establishment of *C. introflexus* . However, the strong association of this species with short time since fire suggests that it may later become outcompeted.

The association between pleurocarpous mosses and a moderately to very long time since fire could be attributed to the prevalence of these mosses in dry moor habitats, often linked to high wildfire severity. Severe wildfires can leave the ground dry and inhospitable for numerous moorland species, leaving it bare for an extended period. Alternatively, the area might become colonized by acrocarpous mosses like *C. introflexus* .

Lichens were associated with long periods since fire, though we did not record individual species (*Cladonia* was the most abundant genus). Thus, we cannot draw conclusions on post‐wildfire succession and lichen diversity. However, our findings align with research on prescribed fire, suggesting lichen richness may peak around 15 years post‐fire (Davies and Legg 2008). Extreme wildfire may promote certain lichens that colonize bare peat, forming a hard crust that hinders the establishment of other species (Maltby et al. 1990). Wildfire effects on moorland lichens are evidently complex, varying with disturbance severity, pre‐fire conditions, and species differences.

Although soil bulk density, pH, C:N ratio, elevation, and slope were significant predictors of vegetation composition, only bulk density showed a relationship with years since fire. Burning and high temperatures have been shown to increase the bulk density of soil (Granged et al. 2011; Stoof et al. 2010), and in addition, a severe fire can remove all or parts of the topsoil and expose deeper soil layers with higher density (Johnstone and Chapin 2006). Increased fire severity and exposure of mineral soil layers can allow for a better seedbed for some species and may, for example, promote the establishment of acrocarpous mosses and forbs (Bret‐Harte et al. 2013; Mallik et al. 2010). We found no evidence of an effect of grazing on species composition or rates of post‐fire regeneration (Hulme et al. 2002), which contrasts with our prediction. This is surprising, as grazing often has a strong impact on regenerating moorland vegetation (Pakeman et al. 2003). However, the result is most likely explained by insufficiently detailed grazing animal density data and does not necessarily reflect real ecological patterns. The data we used only included recent grazing density, and historical values at the time of many of the wildfires may have differed. The data were also on a relatively large spatial scale, and grazing animals are known to be selective and prefer grassland over heathland (Hobbs and Gimingham 1987). More precise information about grazing densities is difficult to acquire but would improve the analyses.

Our results are significant in the context of ongoing biodiversity changes in Scottish moorlands driven by factors like climate change, air pollution, and land use changes (Britton et al. 2009, 2017; Ross et al. 2012). Markedly, the plant species and genera influenced by wildfire align with those associated with continuous biodiversity loss and homogenization. Most notably, Scottish moorland ecosystems have seen a long‐term decrease in lichens and some mosses, for example, *R. lanuginosum* and *Sphagnum* spp., alongside an increase in *C. introflexus* , graminoids, and species that favor higher soil pH (Britton et al. 2017; Pearce and Van Der Wal 2002; Ross et al. 2012). These findings emphasize the importance of considering how wildfire interacts with other environmental changes to shape biodiversity patterns.

### 4.3 Wildfire Effects on Diversity

Contrary to our predictions and previous studies indicating increasing alpha diversity and declining beta diversity over time in blanket bogs (Benscoter and Vitt 2008), we found no significant effect of time since fire on plant species diversity. In the wildfire sites we studied, fires in dry moorland habitats were associated with a reduction in alpha diversity, whereas fires in wet habitats led to a slight increase in alpha diversity. In contrast, beta diversity was influenced solely by fire severity, with higher severity fires having a positive effect.

The effect of habitat on alpha diversity may be related to differences in vegetation composition and microtopography, in addition to the generally different fire severity. The low‐ or moderate‐severity wildfires common in wet moorlands may remove parts of the existing vegetation (e.g., shrubs and graminoids), while leaving others (e.g., the *Sphagnum* layer). This creates space for new species to colonize, while retaining some pre‐fire species. If the new species are similar across the wildfire site, this would lead to an increase in alpha diversity without increasing beta diversity. In contrast, dry moorland habitats, which tend to be more homogeneous and have more combustible moss layers, are likely to burn more evenly and at higher severity (Davies et al. 2016, 2023; Grau‐Andrés et al. 2018). This more severe and uniform burn may remove all existing vegetation, ultimately reducing alpha diversity.

The positive effect of fire severity on beta diversity may be explained by sporadic factors. In areas affected by high‐severity fire, which destroys the existing seedbank and rhizomes (Schimmel and Granström 1996), recolonizing vegetation is more likely to be influenced by random processes, such as where wind‐dispersed propagules land. This randomness can result in high variability within a burnt area, leading to increased beta diversity.

Unlike previous studies showing increasing alpha diversity and declining beta diversity over time in blanket bog (Benscoter and Vitt 2008), we found no effect of time since fire on diversity. This unexpected result may stem from high inter‐ and intra‐site variation or limited sample size, especially in dry moorland. The low fixed‐effects *R* ^2^ and high conditional *R* ^2^ suggest unmeasured site‐specific factors, such as biotic or abiotic conditions and management practices (e.g., rotational burning, drainage, or restoration). Our findings highlight the need for further research to understand the drivers of biodiversity responses to wildfire.

## 5 Conclusions

Our findings offer important insights into moorland wildfire impacts, emphasizing the role of fire severity and the distinctions between dry and wet moor habitats. Wet moors demonstrated resistance to severe burning, with mild effects on vegetation composition, whereas dry moors initially showed strong effects but exhibited recovery over time. After low to moderate severity fires, vegetation composition in burnt areas resembled undisturbed adjacent areas after approximately 20 years. However, high‐severity fires led to greater vegetation change, with regeneration potentially taking longer. The increased risks and consequences of wildfire under climate change may be most severely felt on dry moorland habitats, although extreme weather conditions are also likely to make wet moorlands more susceptible to severe fires and vegetation change. Colonization by the non‐native moss *Campylopus introflexus* was linked to recent and severe burns, particularly in wet moorlands, and may hinder keystone species establishment. Although the important peat‐forming genus Sphagnum exhibited resilience to fire, managing and restoring blanket bogs is crucial for its optimal growth amid climate change and increasing fire severity, ensuring long‐term peatland function.

## Author Contributions

**Noemi A. L. Naszarkowski:** conceptualization (equal), formal analysis (equal), investigation (equal), methodology (equal), project administration (equal), visualization (lead), writing – original draft (lead). **Sarah J. Woodin:** conceptualization (equal), funding acquisition (supporting), investigation (supporting), methodology (equal), project administration (supporting), supervision (lead), validation (equal), writing – review and editing (equal). **Louise C. Ross:** conceptualization (equal), formal analysis (supporting), methodology (equal), supervision (supporting), writing – review and editing (supporting). **Alison J. Hester:** conceptualization (equal), formal analysis (supporting), methodology (equal), supervision (supporting), validation (equal), writing – review and editing (supporting). **Robin J. Pakeman:** conceptualization (lead), formal analysis (supporting), funding acquisition (lead), investigation (supporting), methodology (equal), project administration (supporting), supervision (lead), writing – review and editing (equal).

## Conflicts of Interest

The authors declare no conflicts of interest.

## Supporting information


Data S1

## Data Availability

All relevant data associated with this paper are available in the Supporting Information.

## Appendix 3 Results of a Linear Mixed Model Assessing Factors Influencing Bray–Curtis Dissimilarity Between Burnt and Unburnt Quadrats, Compared to the Unburnt Reference, Using Elevation and Slope Instead of Habitat. This Model had an Akaike Information Criterion Corrected for Small Sample Sizes (AICc) of −385, With a Fixed‐Effect *R* ^2^ of 0.24 and a Conditional *R* ^2^ of 0.54. The Table Presents Standardized Coefficient Estimates (±95% Confidence Intervals), *T*‐Values, and *P*‐Values. Predictor Variables Include Years Since Fire, Elevation (M), Slope (Degrees), Grazing (Log‐Transformed Livestock Units), and Fire Severity (Measured as Difference Normalized Burn Ratio, dNBR).

**Table:**

	Estimate	±95% CI	*t*	*p*
Years	−0.107	0.073	−2.882	0.008
Elevation	0.090	0.081	2.196	0.035
Slope	−0.030	0.035	−1.709	0.089
Grazing	−0.054	0.067	−1.569	0.124
Severity	0.084	0.044	3.748	< 0.001

## Appendix 6 Results of a Linear Mixed Model Assessing Factors Influencing Shannon Diversity of Burnt Quadrats, Compared to the Unburnt Reference, Using Elevation and Slope Instead of Habitat. This Model had a Small‐Sample Corrected Akaike Information Criterion (AICc) of 286, With a Fixed‐Effect *R* ^2^ of 0.11 and a Conditional *R* ^2^ of 0.32. The Table Presents Standardized Coefficient Estimates (±95% Confidence Intervals), *T*‐Values, and *P*‐Values. Predictor Variables Include Years Since Fire, Elevation (M), Slope (Degrees), Grazing (Log‐Transformed Livestock Units), and Fire Severity (Measured as Difference Normalized Burn Ratio, dNBR).

**Table:**

	Estimate	±95% CI	*t*	*p*
Years	0.063	0.181	0.680	0.504
Elevation	−0.270	0.214	−2.482	0.018
Slope	0.111	0.111	1.968	0.050
Grazing	0.086	0.181	0.936	0.356
Severity	−0.101	0.134	−1.470	0.144
